# Impact of sex and *APOE* ε4 on age-related cerebral perfusion trajectories in cognitively asymptomatic middle-aged and older adults: A longitudinal study

**DOI:** 10.1177/0271678X211021313

**Published:** 2021-06-08

**Authors:** Rui Wang, Jennifer M Oh, Alice Motovylyak, Yue Ma, Mark A Sager, Howard A Rowley, Kevin M Johnson, Catherine L Gallagher, Cynthia M Carlsson, Barbara B Bendlin, Sterling C Johnson, Sanjay Asthana, Laura Eisenmenger, Ozioma C Okonkwo

**Affiliations:** 1 Wisconsin Alzheimer’s Disease Research Center, University of Wisconsin School of Medicine and Public Health, Madison, WI, USA; 2 The Swedish School of Sport and Health Science, GIH, Stockholm, Sweden; 3 Division of Clinical Geriatrics, Department of Neurobiology, Care Sciences and Society, Karolinska Institutet and Stockholm University, Stockholm, Sweden; 4 Geriatric Research Education and Clinical Center, William S. Middleton Memorial Veterans Hospital, Madison, WI, USA; 5 Wisconsin Alzheimer’s Institute, University of Wisconsin School of Medicine and Public Health, Madison, WI, USA; 6 Department of Radiology, University of Wisconsin School of Medicine and Public Health, Madison, WI, USA; 7 Department of Medical Physics, University of Wisconsin School of Medicine and Public Health, Madison, WI, USA

**Keywords:** Cerebral perfusion, Alzheimer’s disease, chromosomal sex, APOE gene, cardiometabolic measurements

## Abstract

Cerebral hypoperfusion is thought to contribute to cognitive decline in Alzheimer’s disease, but the natural trajectory of cerebral perfusion in cognitively healthy adults has not been well-studied. This longitudinal study is consisted of 950 participants (40—89 years), who were cognitively unimpaired at their first visit. We investigated the age-related changes in cerebral perfusion, and their associations with *APOE-*genotype, biological sex, and cardiometabolic measurements. During the follow-up period (range 0.13—8.24 years), increasing age was significantly associated with decreasing cerebral perfusion, in total gray-matter (β=−1.43), hippocampus (−1.25), superior frontal gyrus (−1.70), middle frontal gyrus (−1.99), posterior cingulate (−2.46), and precuneus (−2.14), with all P-values < 0.01. Compared with male-ɛ4 carriers, female-ɛ4 carriers showed a faster decline in global and regional cerebral perfusion with increasing age, whereas the age-related decline in cerebral perfusion was similar between male- and female-ɛ4 non-carriers. Worse cardiometabolic profile (i.e., increased blood pressure, body mass index, total cholesterol, and blood glucose) was associated with lower cerebral perfusion at all the visits. When time-varying cardiometabolic measurements were adjusted in the model, the synergistic effect of sex and *APOE*-ɛ4 on age-related cerebral perfusion-trajectories became largely attenuated. Our findings demonstrate that *APOE*-genotype and sex interactively impact cerebral perfusion-trajectories in mid- to late-life. This effect may be partially explained by cardiometabolic alterations.

## Introduction

Emerging evidence has revealed that cerebral perfusion, measured by arterial spin labeling magnetic resonance imaging (ASL-MRI), is a non-invasive biomarker that may capture an upstream feature of AD neuropathology,^1,2^ and may inform both disease risk and physiological changes of the aging brain.^3^ Although the cause remains unclear, age-related reduction in ASL perfusion has been reported by previous studies involving cognitively intact individuals.^4^ Most studies have demonstrated reduced average cerebral perfusion in gray matter with advancing age, but controversial findings exist in regional variations of cerebral perfusion reduction.^5–10^ Previous research either contrasted ASL perfusion between young and older age groups dichotomously,^5–8^ or used a cross-sectional design involving individuals with a wide range of age (e.g., 20—80 years).^9,10^ Little research has been done on the natural trajectory of ASL perfusion with age in functionally intact middle-aged and older adults.

Besides old age, *APOE* ε4 allele and chromosomal female sex are two well-established unmodifiable factors that increase the risk of late-onset AD.^11,12^ Consistent with that finding, evidence indicates that among patients with cognitive impairment, regional cerebral perfusion deficits differ by sex and *APOE* ε4 status.^13–15^ Nevertheless, it remains unknown, whether *APOE* ε4 allele and female sex could, independently or synergistically, modify cerebral perfusion trajectories with increasing age in asymptomatic middle-aged and older adults. In addition, cardiometabolic risk factors, such as high levels of blood pressure and cholesterol, have been established as important modifiable risk factors for AD. Furthermore, these factors appear to interact with *APOE* ε4 status and sex to increase AD risk.^16,17^ Thus, we also sought to clarify the role of cardiometabolic measurements in the patterns of cerebral perfusion trajectories with increasing age.

Within two longitudinal cohorts of middle-aged and older adults (40-89 years old), we analyzed serial ASL perfusion measures in AD-vulnerable regions to determine 1) the association between cerebral perfusion trajectories and age; 2) the modifying effect of *APOE* ε4 status and sex on the relationship between cerebral perfusion trajectories and age; 3) the role of cardiometabolic measurements in the foregoing associations. We hypothesized that cerebral perfusion decreases with increasing age; that *APOE* ε4 carriage and female sex synergistically lead to further deterioration in cerebral perfusion; and that cardiometabolic health is related to cerebral perfusion trajectory, and partially mediates the *APOE* ε4 and sex effect.

## Material and methods

### Participants

Data for this report came from 950 cognitively unimpaired individuals enrolled in two ongoing longitudinal cohorts, the Wisconsin Registry for Alzheimer’s Prevention (WRAP) and the Wisconsin Alzheimer’s Disease Research Center (WADRC), between November 24, 2009, and August 3, 2018.^18,19^ To be included in this report, participants were required to: a) be cognitively intact and without stroke or other severe neurological disorder; and b) have at least one ASL scan, in addition to meeting standard WRAP/WARDC enrollment criteria which include being 40–65 years at baseline, fluent English speaker, visual and auditory acuity adequate for neuropsychological testing, and overall good health with no diseases expected to interfere with study participation over time.^13,18^ Of the 950 individuals, 537 had two visits, 255 had a third visit, 151 had a fourth visit, and 47 had five or more visits. The average follow-up time was 2.76 years (median: 2.17 years, interquartile range [IQR]: 1.51-3.99 years, range: 0.13-8.24 years). In total, after excluding 35 scans with poor neuroimaging quality, 1940 scans were available for analysis.

The data collection was approved by the University of Wisconsin Institutional Review Board and within the guidelines of the Helsinki Declaration. Written informed consent was provided by each participant.

### Demographic factors and cardiometabolic measurements

Age was collected on the day of MRI scan acquisition as a continuous variable with two decimals. Sex was reported as woman or man. Educational level was defined according to the maximum years of formal schooling. At each study visit, cardiometabolic measurements, i.e., systolic blood pressure, blood glucose, total cholesterol, weight, and height were measured by physical examination or laboratory test at the University of Wisconsin Clinical Research Unit.^18^ Blood glucose and total cholesterol level were measured from blood drawn after a minimum 12-hour overnight fast.^20^ Using a random-zero sphygmomanometer with individualized cuff size, blood pressure was measured up to three times (to ensure stability of readings) with the participant in a seated position.^21^ Body mass index (BMI) was calculated as weight (kilogram) divided by squared height (meter).

### *APOE* genotyping

Determination of *APOE* genotype has been described previously.^18,22^ We classified our participants into two groups: *APOE* ε4 carriers (one or more ε4 alleles present) or *APOE* ε4 non-carriers (no ε4 allele present).

### Neuroimaging protocol

MRI data were acquired on two identical clinical 3 T scanners (Discovery MR750, General Electric, Waukesha, WI, USA),^20^ and 1642 scans were acquired using an 8-channel head coil (Excite HD Brain Coil; GE Healthcare) whereas 298 scans were acquired using a 32-channel head coil (Nova Medical). We collected 3 D T1-weighted inversion recovery-prepared spoiled gradient echo scans with the following parameters: inversion time (T1)=450 ms, echo time (TE) = 3.2 ms, repetition time (TR)=8.2 ms; flip angle = 12°, slice thickness =1.0 mm, field of view (FOV) = 256 mm, acquisition matrix = 256 × 256.

Cerebral perfusion was measured using background-suppressed pseudocontinuous ASL (pcASL) MRI,^23^ utilizing a 3 D fast spin-echo stack of spiral sequence. Scan parameters were as follows: echo spacing = 4.9 ms; TE = 10.5 ms with centric phase encoding; spiral arms = 8, spiral readout duratio =4ms, FOV = 240×240×176mm; 4 mm isotropic spatial resolution; reconstructed matrix size = 128×128×44; number of averages (NEX) = 3; and labeling RF amplitude = 0.24×mG, scan time = 4.5 minute. Immediately after each ASL scan, a proton density (PD) reference scan was performed with identical imaging acquisition parameters without ASL labeling but with a saturation pulse was applied 2.0 seconds prior to imaging. This PD image was used for ASL flow quantification as well as for imaging registration.^13^

To improve signal-to-noise ratio, we averaged the three excitations that comprise the pcASL sequence (i.e., NEX = 3). The entire pcASL sequence, including all 3 excitations and PD scan, took 4.5 minutes. An excellent test-retest reliability (rcorrelation > 0.95) for this pcASL procedure has been reported previously.^23^ Cerebral perfusion is reported in ml/100g/min units. In the present analytical sample, intra-class correlation coefficient for repeated cerebral perfusion in global gray matter was 0.97 (95% confidence interval [CI]: 0.80 to 0.99).

Also because of protocol changes, post-labeling delay was 2025 ms for 79% of scans and 1525 ms for the rest. To account for potential heterogeneity arising from the foregoing protocol changes, head coil and post-labeling delay were included as covariates in our analyses.^13^ Furthermore, in sensitivity analyses we excluded all scans with 1525 ms post-labeling delay to minimize potential measurement bias of this factor on cerebral perfusion quantitation.

### ASL processing

**Figure 1.**
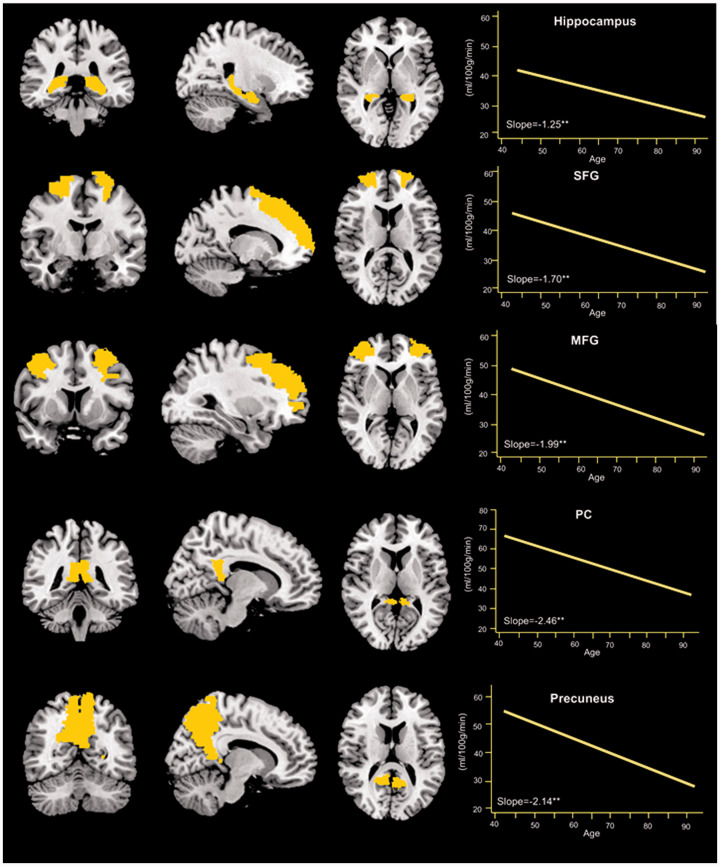
Average slope of regional cerebral perfusion changes with age (n = 950). SFG: Superior frontal gyrus; MFG: middle frontal gyrus; PC: posterior cingulate. *Note*. The brain slices represent coronal, sagittal, and axial views of the *a priori* mask for each region. The graphs represent the average slope of cerebral perfusion in each region in relation to age. **p<0.01.

Measures were extracted from pcASL cerebral perfusion images using SPM12 tool (http://www.fil.ion.ucl.ac.uk/spm/software/spm12/). Each participant’s PD image was first registered to the T1 image, and then the derived transformation matrix was applied to the average quantitative cerebral perfusion map. With resampling to a 2x2x2 mm^3^ voxel size, the T1 volume and associated cerebral perfusion image were subsequently spatially normalized to the Montreal Neurological Institute (MNI) template. The normalized cerebral perfusion maps were then smoothed using an 8-mm full-width at half-maximum Gaussian kernel. To reduce the risk of false-positive errors and focus our analyses on brain regions that are known to be critical in AD we imposed an a priori anatomical mask (Figure 1) that included the hippocampus, superior frontal gyrus, middle frontal gyrus, posterior cingulate, and precuneus using the WFU PickAtlas toolbox.^24^ Our previous ASL work in asymptomatic middle-aged adults with maternal history of Alzheimer’s disease showed reduced cerebral perfusion in these regions.^13^ We also examined total gray matter perfusion.

### Statistical analysis

Characteristics of study participants at their first MRI scan, by *APOE* ε4 status and sex, were compared using Chi-square test for proportions and t-test for means. Continuous variables that were not normally distributed were examined using Wilcoxon signed-rank test. With age as the time scale, we used mixed-effects models to explore the longitudinal changes in cerebral perfusion. Covariates included in the fully adjusted model were birth cohort (defined by year of birth), sex, *APOE* ε4 status, education, parental history of dementia, smoking status, intracranial volume, post-labeling delay, and head coil. Certain covariates (e.g., education level, parental history of dementia, smoking status) had missing data.

Both random intercept and random slope were considered in the models, and an unstructured covariance structure was implemented given its flexibility and generalizability when there is no *a priori* formulation of the functional form of the data.^25^ To test the modifying effect of *APOE* ε4 status and sex on age-related cerebral perfusion trajectories, we included interaction terms for *APOE* ε4×age, sex×age, and *APOE* ε4×sex×age, in the mixed-effects models. The association between cardiometabolic measurements and cerebral perfusion trajectories was estimated in the mixed-effects models by calculating the β-coefficients and 95% CI of time-varying cardiometabolic measurements, i.e., systolic blood pressure, body mass index, total cholesterol, and blood glucose. Likelihood-ratio tests between nested models were performed to estimate the contribution of time-varying cardiometabolic measurements to age-related cerebral perfusion trajectories.

To further assess the robustness of our findings, we conducted the following sensitivity analysis: 1) we excluded those with follow-up time less than 3 months as well as those who had incident cognitive impairment, 2) we excluded scans with 1525 ms post-labeling delay, 3) we re-ran the analyses after applying a locally-derived correction factor of 1.3669 to all scans collected using a 2025 ms post-labeling delay cerebral perfusion; and 4) only included those who had at least two MRI scans. In additional analyses, we tested the possibility of a nonlinear relationship between cerebral perfusion changes and age by including the quadratic effect of age in the models. Stata 14.0 for Windows (StataCorp., College Station, TX, USA) was used for all analyses. Only findings that met an alpha threshold of 0.05 were deemed significant.

## Results

### Background characteristics of study participants

The characteristics of study participants at the first visit, by *APOE* ε4 status and sex, are shown in Table 1. The average age was 60.29 (Standard Deviation [SD] 7.75) years, and 38.52% were *APOE* ε4 carriers. There were no gender differences across *APOE* ε4 strata: ε4 carriers were composed of 67.47% women compared to 66.96% among non-carriers. No differences were observed in age, parental history of dementia, race, body mass index, or smoking status by *APOE* ε4 status and sex. Compared with male ε4 non-carriers, female ε4 non-carriers showed lower levels of education, blood pressure, blood glucose, and intracranial volume. Conversely, they had higher levels of total cholesterol and cerebral perfusion (all regions except the hippocampus). Similarly, compared with male ε4 carriers, female ε4 carriers had lower levels of education, diastolic blood pressure, and intracranial volume, but had higher levels of total cholesterol and cerebral perfusion.

**Table 1.** Participant characteristics at the magnetic resonance imaging phase first visit.

	Total sample(n = 950)	*APOE* ε4 non-carriers (n = 541)^a^			*APOE* ε4 carriers (n = 339)^a^		
		Men(n = 176)	Women(n = 365)	*p*-Value	Men (n = 112)	Women(n = 227)	*p*-Value
Age (year), mean (SD)	60.29 (7.75)	61.50 (7.56)	61.01 (7.46)	0.479	60.52 (7.72)	59.65 (7.94)	0.343
Age group (years), n (%)							
40–49	78 (8.21)	11 (6.23)	24 (6.58)		11 (9.82)	18 (7.93)	
50–59	395 (41.58)	63 (35.80)	139 (38.08)		43 (38.39)	113 (49.78)	
60–69	382 (40.21)	82 (46.59)	163 (44.66)		48 (42.86)	71 (31.28)	
70–79	79 (8.32)	16 (9.09)	33 (9.04)		8 (7.14)	21 (9.25)	
≥80	16 (1.68)	4 (2.27)	6 (1.64)	0.528	2 (1.79)	4 (1.76)	0.374
Education (year), mean (SD)^a^	16.11 (2.31)	16.64 (2.57)	15.85 (2.25)	<0.001	16.75 (2.23)	15.81 (2.12)	<0.01
Parental history of dementia, n (%)^a^	578 (65.61)	100 (57.47)	213 (58.52)	0.818	84 (75.68)	179 (78.85)	0.509
Race, n (%)^a^							
White	791 (89.78)	157 (90.23)	330 (90.66)		103 (92.79)	199 (87.67)	
Black/African American	68 (7.72)	14 (8.05)	24 (6.59)		6 (5.41)	21 (9.25)	
Other	22 (2.50)	3 (1.71)	10 (2.74)	0.408	0 (0.00)	7 (3.08)	0.357
Blood pressure (mmHg), mean (SD)^a^							
Systolic blood pressure	126.08 (16.18)	129.79 (14.99)	124.86 (16.82)	0.001	127.37 (13.21)	124.52 (17.03)	0.123
Diastolic blood pressure	75.45 (9.32)	78.98 (8.75)	73.68 (9.18)	<0.001	78.37 (9.21)	73.95 (8.89)	<0.001
Body mass index (kg/m^2^), mean (SD)^a^	28.44 (5.76)	28.55 (4.09)	28.25 (6.23)	0.563	28.10 (4.29)	28.80 (6.61)	0.305
Blood Glucose(mg/dL), mean (SD)^a^	98.96 (20.91)	104.79 (23.99)	98.56 (23.80)	0.006	98.63 (13.21)	95.36 (14.81)	0.058
Total cholesterol (mg/dL)^a^	198.14 (37.67)	186.11 (38.81)	201.60 (36.85)	<0.001	189.68 (38.36)	206.02 (35.00)	<0.001
Ever smoked, n (%)^a^	343 (38.84)	61 (35.06)	150 (41.21)	0.172	35 (31.53)	96 (42.29)	0.057
Intracranial volume (mL), mean (SD)	1473.42 (143.15)	1603.12 (122.76)	1407.89 (96.71)	<0.001	1619.75 (121.71)	1402.13 (94.27)	<0.001
Cerebral perfusion (mL/100 g/min), Mean (SD)							
Total gray matter	37.08 (11.06)	34.50 (11.04)	37.71 (11.77)	0.003	33.36 (8.24)	39.27 (10.85)	<0.001
Hippocampus	38.58 (12.83)	37.27 (12.81)	38.65 (14.17)	0.278	36.35 (10.59)	40.08 (12.30)	0.006
Superior frontal gyrus	40.38 (13.08)	37.65 (14.86)	41.18 (13.17)	0.005	35.64 (9.14)	42.87 (12.72)	<0.001
Middle frontal gyrus	43.92 (14.00)	40.20 (15.22)	45.07 (13.91)	<0.001	38.09 (9.75)	47.00 (13.90)	<0.001
Posterior cingulate	58.94 (21.20)	54.82 (20.73)	60.20 (22.60)	0.008	52.69 (17.84)	62.15 (20.58)	<0.001
Precuneus	46.44 (16.16)	41.67 (16.25)	47.93 (16.94)	<0.001	40.33 (12.04)	50.01 (15.66)	<0.001

SD: standard deviation.

*p*-Value was obtained using Chi-square test for categorical variables and *t*-test for continuous variables. If the continuous variable was not normally distributed, Wilcoxon signed-rank test was applied.

^a^Missing value: 70 for *APOE* ε4 status, 69 for education, 69 for parental history of dementia, 69 for race, 69 for blood pressure and body mass index, 128 for blood glucose, 67 for serum total cholesterol, 67 for smoking status. Because the missing value was less than 10%, we imputed the missing value as either a dummy variable (for discrete variables) or with their mean value (for numerical variable) when those variables were controlled as covariates in further analyses.

### Age-related cerebral perfusion trajectories

After controlling for birth cohort, sex, *APOE* ε4 status, education year, parental history of dementia, smoking status, intracranial volume, post-labeling delay, and head coil in the fully adjusted models, we found a linear relationship between increasing age (in 5-year increments) and cerebral perfusion reduction in total gray matter (β [95% CI] =−1.43 [−1.79 to −1.07]), hippocampus (−1.25 [−1.70 to −0.80]), superior frontal gyrus (−1.70 [−2.18 to −1.22]), middle frontal gyrus (−1.99 [−2.52 to −1.46]), posterior cingulate (−2.46 [−3.26 to −1.67]), and precuneus (−2.14 [−2.76 to −1.52]) (Table 2 and Figure 1). Similarly, when age was treated as a categorical variable in the models, the results showed that compared with the quadragenarians, other age groups all showed significant reduction in cerebral perfusion across all the brain regions (Table 2).

**Table 2.** Association of cerebral perfusion with age, sex, and *APOE* ε4 status (n = 950).

	Total gray matter^a^	Hippocampus^a^	Superior frontal gyrus^a^	Middle frontal gyrus^a^	Posterior cingulate^a^	Precuneus^a^
Linear model, 5-yrs						
Age, 5-year	−1.43** (−1.79, −1.07)	−1.25** (−1.70, −0.80)	−1.70** (−2.18, −1.22)	−1.99** (−2.52, −1.46)	−2.46** (−3.26, −1.67)	−2.14** (−2.76, −1.52)
Categorical age						
Age groups						
40–49	Ref.	Ref.	Ref.	Ref.	Ref.	Ref.
50–59	−3.10** (−5.03, −1.18)	−3.18** (−5.13, −1.23)	−2.89** (−5.00, −0.78)	−3.26** (−5.57, −0.95)	−5.76** (−9.16, −2.36)	−4.95** (−7.63, −2.26)
60–69	−4.72** (−6.87, −2.57)	−4.42** (−6.72, −2.13)	−4.88** (−7.37, −2.38)	−5.62** (−8.35, −2.90)	−7.99** (−12.00, −3.99)	−7.40** (−10.57, −4.23)
≥70	−7.10** (−9.64, −4.58)	−6.37** (−9.25, −3.49)	−7.12** (−10.26, −3.99)	−8.23** (−11.66, −4.81)	−11.68** (−16.66, −6.69)	−10.46** (−14.43, −6.50)
*APOE* ε4						
Age× *APOE* ε4	0.21 (−0.36, 0.79)	0.05 (−0.61, 0.71)	0.08 (−0.62, 0.79)	0.11 (−0.66, 0.87)	0.00 (−1.16, 1.17)	0.20 (−0.71, 1.11)
Sex						
Age×Sex	−0.37 (−0.95, 0.20)	−0.11 (−0.75, 0.54)	−0.53 (−1.23, 0.16)	−0.65 (−1.41, 0.11)	−0.64 (−1.80, 0.51)	−0.82 (−1.72, 0.07)
*APOE* ε4 and sex						
Age×ε4×Sex	−1.23* (−2.46, −0.03)	−1.52* (−2.98, −0.12)	−1.61* (−3.10, −0.11)	−1.85* (−3.49, −0.21)	−1.96 (−4.45, 0.52)	−1.70 (−3.64, 0.24)

^a^The β-coefficients and 95% confidence intervals in the models were adjusted for birth cohort, sex, *APOE* ε4 status, education, parental history of dementia, smoking status, intracranial volume, post-labeling delay, and head coil.

*0.01<*p* < 0.05; **p < 0.01.

### The modifying effect of *APOE* ε4 status and sex on cerebral perfusion trajectories

**Figure 2.**
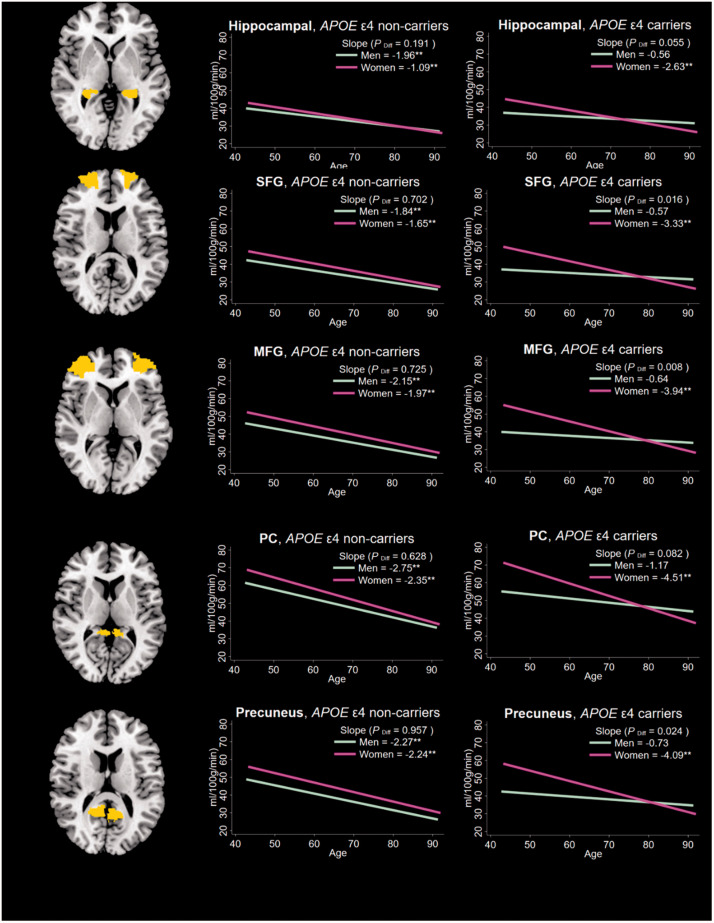
Average slope of regional cerebral perfusion change with age by sex and *APOE* ε4 status (n = 880). SFG: superior frontal gyrus; MFG: middle frontal gyrus; PC: posterior cingulate. *Note*. The brain slices are sagittal views of the *a priori* mask for each region. The left graphs represent the average slope of cerebral perfusion with age in each region for female ε4 non-carriers versus male ε4 non-carriers, whereas the right graphs represents the average slope of cerebral perfusion with age in each region for female ε4 carriers versus male ε4 carriers. *P*_diff_: *p*-value for the test for a statistical difference between the slope for men versus women. ^**^p<0.01.

In the fully adjusted model, we observed a three-way interactive effect of age, *APOE* ε4, and sex on the cerebral perfusion trajectories in total gray matter (P = 0.043), hippocampus (P = 0.038), superior frontal gyrus (P = 0.033), middle frontal gyrus (P = 0.021), and precuneus (P = 0.071). To further investigate this three-way interaction, we classified participants into four groups by *APOE* ε4 status and sex. The slope of total gray matter cerebral perfusion change with every 5-year increase in age was −0.55 (P = 0.234) for male ε4 carriers, −2.74 (P < 0.01) for female ε4 carriers, −1.60 (*P* < 0.01) for male ε4 non-carriers, and −1.37 (P < 0.01) for female ε4 non-carriers. This gender differential in slope was not significant among ε4 non-carriers (P = 0.594) but was significant among ε4 carriers (P = 0.027). Similar patterns of age-related cerebral perfusion trajectories by *APOE* ε4 status and sex were seen in the regions of interest (Figure 2).

In order to further understand the different rates of age-related decline in cerebral perfusion between female and male ε4 carriers, we plotted the average decline rates in cerebral perfusion by *APOE* zygosity (homozygotes versus heterozygotes) (Supplementary Figure 1). The results showed that in male ε4 carriers, homozygous individuals (i.e., ε4/ε4) presented faster cerebral perfusion decline with age in total gray matter, superior frontal gyrus, middle frontal gyrus, posterior cingulate, and precuneus, than the heterozygotes (i.e., ε2/ε4 or ε3/ε4). In contrast, among female ε4 carriers, there was no difference in age-related cerebral perfusion decline as a function of *APOE* zygosity. This suggests that among female ε4 carriers, with advancing age, biological sex is a stronger determinant of cerebral perfusion decline than mere ε4 zygosity whereas the reverse is true among their male counterparts.

### The role of cardiometabolic measurements in the age-related trajectories

Poor cardiometabolic health was associated with decreasing cerebral perfusion over time (Supplementary Table 1). Because cardiometabolic indices differed as a function of *APOE* ε4 status and sex (Table 1) we hypothesized that change in cardiometabolic measurements may explain, at least partly, the effects of *APOE* ε4 status and sex on age-related cerebral perfusion trajectories.

Because there were no differences in age-related cerebral perfusion decline between female and male ε4 non-carriers (see Figure 2), for these set of interrogations, we collapsed them into one group resulting in three groups of participants (i.e., ε4 non-carriers, male ε4 carriers, and female ε4 carriers). In the fully adjusted model, compared with male ε4 carriers who showed the slowest cerebral perfusion slope, female ε4 carriers showed the fastest rate of age-related decline followed by ε4 non-carriers (Table 3). These associations became largely attenuated after adding time-varying cardiometabolic covariates to the model, i.e., systolic blood pressure, glucose, body mass index, and total cholesterol. The results of the likelihood-ratio tests between the two models confirmed the significant contributions of the cardiometabolic variables across all the examined brain regions (*P* < 0.001).

**Table 3.** The effect of time-varying cardiometabolic measurements on sex- and *APOE* ε4-related cerebral perfusion decline with age (n = 880).^a^

	Gray matter^a^	Hippocampus^a^	Superior frontal gyrus^a^	Middle frontal gyrus^a^	Posterior cingulate^a^	Precuneus^a^
Model 1^b^						
Men ε4 carriers	Ref (0.00)	Ref (0.00)	Ref (0.00)	Ref (0.00)	Ref (0.00)	Ref (0.00)
ε4 non-carriers	−0.83 (−1.78, 0.12)	−0.68 (−1.72, 0.35)	−1.10 (−2.20, 0.01)	−1.31 (−2.52, −0.10)*	−1.20 (−3.05, 0.65)	−1.40 (−2.84, 0.05)
Women ε4 carriers	−1.14 (−2.20, −0.09)*	−0.94 (−2.08, 0.21)	−1.57 (−2.79, −0.34)*	−1.82 (−3.15, −0.48)**	−1.77 (−3.81, 0.27)	−1.76 (−3.35, −0.16)*
Model 2						
Model 1+time-varying covariates of cardiometabolic measurements^c^						
Men ε4 carriers	Ref (0.00)	Ref (0.00)	Ref (0.00)	Ref (0.00)	Ref (0.00)	Ref (0.00)
ε4 non-carriers	−0.79 (−1.80, 0.23)	−0.69 (−0.42, 8.72)	−0.84 (−2.06, 0.39)	−1.04 (−2.37, 0.29)	−1.28 (−3.24, 0.68)	−1.43 (−3.00, 0.15)
Women ε4 carriers	−0.94 (−2.07, 0.19)	−0.85 (−2.06, 0.36)	−1.30 (−2.67, 0.07)	−1.51 (−2.99, −0.02)*	−1.51 (−3.70, 0.68)	−1.63 (−3.39, 0.13)
Likelihood-ratio test between Model 1 and Model 2						
Chi^2^ (*p*-value)	61.52 (<0.001)	40.28 (<0.001)	64.18 (<0.001)	72.34 (<0.001)	46.84 (<0.001)	56.10 (<0.001)
AIC						
Model 1	8710.77	8911.84	9210.33	9404.62	10336.31	9807.97
Model 2	8660.52	8879.62	9157.72	9348.28	10298.4	9763.27

^a^There were 70 participants with missing information on *APOE* ε4 status.

^b^The β-coefficients and 95% confidence intervals in the models were adjusted birth cohort, sex, *APOE* ε4 status, education, parental history of dementia, smoking status, intracranial volume, post-labeling delay, and head coil.

^c^Time-varying covariates were systolic blood pressure, body mass index, blood glucose, and total cholesterol.

*0.01<*p* < 0.05; **p < 0.01.

### Sensitivity analyses

All findings described above were substantively unchanged after excluding those whose follow-up time was less than 3 months and those with incident cognitive impairment. Likewise, in the analyses that applied a correction factor to scans collected with 2025 ms post-labeling delay, those that only included scans with 2025 ms post-labeling delay (Supplementary Table 2), and those that only included individuals who had at least two MRI scans, the original results persisted. When we tested for nonlinearity in the association between age and cerebral perfusion trajectories (Supplementary Table 3), we did not detect substantial findings.

## Discussion

In this longitudinal study, we observed that: 1) among adults aged 40-89 years old, aging is associated with a cerebral perfusion decrease in total gray matter, hippocampus, superior frontal gyrus, middle frontal gyrus, posterior cingulate, and precuneus; 2) *APOE* ε4 allele and female sex synergistically accelerate age-related cerebral perfusion decline in total and regional gray matter; 3) poor cardiometabolic profile (i.e., higher systolic blood pressure, blood glucose, body mass index, and total cholesterol) are related to a reduction in cerebral perfusion over time, which partially explained the effects of *APOE* ε4 status and sex on age-related cerebral perfusion decline.

Although accumulative evidence has emphasized the importance of cerebral perfusion as a potential marker of neurovascular health associated with AD, very few studies have described age-related cerebral perfusion trajectories in cognitively normal adults, especially in a longitudinal manner. Using^15^ O–labeled water positron emission tomography (PET), the Baltimore Longitudinal Study of Aging (BLSA) revealed that in older adults without cognitive impairment (>55 years), cerebral perfusion declines over time, and the rate of decline differs by cardiovascular health, *APOE* ε4 status, and amyloid burden.^26–29^ Specifically, the BLSA reported a faster cerebral perfusion decline over a period of 6-8 years in hypertensive participants than in those who were normotensive,^26^ in participants with impaired glucose tolerance than in participants with normoglycemia, in *APOE* ε4 carriers than in ε4 noncarriers, and in groups with high amyloid deposition than in those with low amyloid deposition. One cross-sectional study of cognitively normal older adults aged 55-85 years, found that ASL-quantified cerebral perfusion in gray matter was negatively correlated with age.^30^ Similarly, another cross-sectional study of healthy adults aged 23-88 years reported age-related ASL perfusion reductions in cortical gray matter areas including superior frontal, orbitofrontal, superior parietal, middle and inferior temporal, insular, precuneus, supramarginal, lateral occipital, and cingulate regions.^9^ To our knowledge, our study is the first attempt to capture the natural trajectory of cerebral perfusion change in a cognitively unimpaired aging population using longitudinal ASL-quantified cerebral perfusion data and focusing on brain regions that are closely related to AD risk, such as hippocampus, posterior cingulate, and the precuneus.^13^ Our data indicate that cerebral perfusion of the posterior cingulate and precuneus declines faster with increasing age than that in other gray matter regions. This is consistent with previous reports that concluded that cerebral perfusion of these two brain structures is closely associated with AD, and can be potentially useful neuroimaging markers to identify mild cognitive impairment (MCI) and AD.^31,32^

MCI and AD patients exhibit greater cerebral hypoperfusion in AD-vulnerable regions than those without cognitive impairment.^13,14^ However, in non-symptomatic adults with a risk profile of dementia/AD, ASL-MRI-assessed cerebral perfusion may display diverse patterns. Specifically, the effect of *APOE* ε4 allele on cerebral perfusion appears to be modified by age, with evidence that older ε4 adults display decreased cerebral perfusion and younger ε4 carriers show increased perfusion.^33–35^ It has been suggested that such a “compensatory” cerebral perfusion increase in younger individuals with AD risk profiles may be an attempt to maintain cognitive function via increased metabolic demands.^33^ The present study showed that although neither *APOE* genotype nor sex individually modified age-related cerebral perfusion decline, they exerted an interactive effect on age-related cerebral perfusion trajectories. Compared with non-ε4 carriers and male ε4 carriers, female ε4 carriers exhibited the fastest cerebral perfusion decline. The results indicate that female sex seemingly amplifies the harmful effect of the *APOE* gene on the brain. This points to a critical and commonly overlooked detail of the link between *APOE* ε4 and AD—it is more pronounced in women than in men.^12^ Although studies that investigate the interactions between *APOE* ε4 status and sex on neuroimaging biomarkers are sparse, consistent findings have revealed that *APOE* ε4 confers greater AD risk in women,^36–38^ and this increased *APOE*-related risk in women is observed in tau pathology, cerebral hypometabolism, altered functional connectivity, and brain atrophy.^39,40^ A recent study by the Mayo group has further confirmed that female ε4 carriers accumulate more tangles and have worse memory than male carriers.^41^

It remains unclear why male ε4 carriers presented with the least age-related cerebral perfusion decline in our study, even relative to ε4 non-carriers. Slightly different from the prevalent view that women who carry copies of the *APOE* ε4 allele have a greater AD risk than men with the same number of copies,^42^ we found that among male ε4 carriers, age-related cerebral perfusion decline appeared faster among homozygotes than heterozygotes. Considering the same rate of age-related cerebral perfusion decline among homozygotes and heterozygotes was detected among female ε4 carriers, our findings propose that during the aging process, compared with mere ε4 zygosity, cerebral perfusion decline was largely driven by biological sex among female ε4 carriers. In contrast, ε4 zygosity was the main determinant among male ε4 carriers. Future studies are necessary to carefully investigate the role of *APOE* ε4 zygosity in the interactive effect of sex and *APOE* ε4 status on brain aging and AD onset.

Links between cardiovascular risk factors (e.g., increased systolic blood pressure and serum cholesterol level) and cerebral perfusion reduction have been disclosed from previous studies.^26,43,44^ Evidence even suggests that reduced global cerebral perfusion may be a valid imaging biomarker for cardiovascular risk.^41^ In the current study, we provided additional evidence by investigating the dynamic association between cardiometabolic profiles and cerebral perfusion trajectories. Our findings showed that increased cardiometabolic measurements were correspondingly related to decreasing cerebral perfusion during the aging process, which also partially accounted for sex- and *APOE* ε4-related cerebral perfusion decline. In this context, it is interesting to note that an ancillary, unreported, analysis of the data revealed that although men had higher (but still broadly normal) systolic blood pressure readings than women at the outset of the study (the average value was 125.1 mmHg for men and 113.5 mmHg for women), women exhibited an accelerated increase in systolic blood pressure over time such that by the time the cohort was in their 80 s, systolic pressures were similar between the sexes (the average value was 134.2 mmHg for men and 135.8 mmHg for women, Supplementary Figure 2).

It was interesting to note that women exhibited higher cerebral perfusion than men at the start of the study but then experienced a steeper rate of decline as they aged. This phenomenon has been reported in other studies.^45,46^ For example, in an [15O]H2O study of cerebral blood flow, Aanerud and colleagues^45^ found that women had significantly higher cerebral blood flow than men in frontal and temporal lobes in younger ages, but these differences disappeared by the time both groups reached age 65. One possible explanation for the faster cerebral perfusion decline in women is that a bioenergetic shift occurs during perimenopausal transition, resulting in a drop of estrogen, prostacyclin, and CO_2_ reactivity during post-menopause which then manifests as decreased cerebral metabolic function and blood flow.^12,45^ Carrying the *APOE* ε4 risk allele further accelerates this age-related reduction in cerebral metabolism and cerebral perfusion. Other evidence also indicates that impairment of mitochondrial energy production may drive metabolic heterogeneity and consequently cause faster cerebral perfusion decline in specific subgroups, such as female ε4 carriers.^47^ It is also possible that the differential escalation in systolic blood pressure noted above among women may have contributed to their accelerated decline in cerebral perfusion later in life. Additional studies are needed for fully understanding these sex effects.

The strengths of the current study include 1) a large, well-characterized sample size, 2) longitudinal ASL scanning, 3) time-varying measurement of cardiometabolic factors, enabling an examination of their association with cerebral perfusion trajectories over time, and 4) use of mixed-effects models that can properly deal with data collection regimens involving intra- and inter-person variation in number of longitudinal follow-up or time interval between visits.

Several potential limitations should be addressed. First, our sample has an overrepresentation of persons with a parental history of dementia (66%). Although this is by design (the WRAP and WADRC cohorts were originally established to study the role of parental history of dementia on prospective risk for dementia), it necessitates some caution when attempting to generalize our findings to the broader population of persons without such a family history. Second, survival bias may exist and result in the maintained participants not fully representing the total population. Third, we did not consider the use of medications (including dosage and duration) when examining the impact of cardiometabolic measurements on cerebral perfusion trajectories, which may underestimate the association between worsened cardiometabolic profiles and age-related cerebral perfusion decline. Fourth, we measured cerebral perfusion using a single delay arterial spin labeling sequence, which is known to be affected by hemodynamic parameters, macrovascular geometry, and arterial transit times. These effects often lead to artificially lower measures, especially in the parietal regions (“last meadows” phenomenon).^48^ It is possible that our finding of preferential hypoperfusion in the posterior cingulate and precuneus might be a methodological artifact considering that we did not have any available cardiac output data adjusted in our models. We recommend that future investigations of perfusion trajectories in normal aging collect important hemodynamic parameters, or apply methods that account for (e.g. multi-delay ASL) or are independent of (e.g. PET) such hemodynamic parameters. Similarly, it would be advantageous for future studies to include monitoring of end-tidal carbon dioxide concentration in order to generate a more comprehensive understanding of the biological mechanisms underlying the heart-brain connection.^49^ In summary, our findings suggest that cerebral perfusion in both global and regional gray matter declines with advancing age, and the decline differs jointly by *APOE* ε4 status and sex. Female *APOE* ε4 carriers exhibit a precipitous age-related cerebral perfusion decline, which is largely explained by cardiometabolic factors, such as systolic blood pressure, glucose, body mass index, and serum total cholesterol. Future studies extending our findings could clarify the mechanisms underlying the observed sex- and *APOE* ε4-specific effect on cerebral perfusion trajectories and guide the way to personalized prevention for AD.

## Supplemental Material

sj-pdf-1-jcb-10.1177_0271678X211021313 - Supplemental material for Impact of sex and *APOE* ε4 on age-related cerebral perfusion trajectories in cognitively asymptomatic middle-aged and older adults: A longitudinal study Click here for additional data file. Supplemental material, sj-pdf-1-jcb-10.1177_0271678X211021313 for Impact of sex and *APOE* ε4 on age-related cerebral perfusion trajectories in cognitively asymptomatic middle-aged and older adults: A longitudinal study by Rui Wang, Jennifer M Oh, Alice Motovylyak, Yue Ma, Mark A Sager, Howard A Rowley, Kevin M Johnson, Catherine L Gallagher, Cynthia M Carlsson, Barbara B Bendlin, Sterling C Johnson, Sanjay Asthana, Laura Eisenmenger and Ozioma C Okonkwo in Journal of Cerebral Blood Flow & Metabolism
