# Supplementary material for: Impact of sex and APOE ε4 on age-related cerebral perfusion trajectories in cognitively asymptomatic middle-aged and older adults: A longitudinal study
Source: J Cereb Blood Flow Metab. 2021 Jun 8;41(11):3016–27. doi: 10.1177/0271678X211021313 (PMC8545048; doi:10.1177/0271678X211021313)
Supplement: sj-pdf-1-jcb-10.1177_0271678X211021313 - Supplemental material for Impact of sex and APOE ε4 on age-related cerebral perfusion trajectories in cognitively asymptomatic middle-aged and older adults: A longitudinal study [file sj-pdf-1-jcb-10.1177_0271678X211021313.pdf]

SUPPLEMENTARY MATERIALS

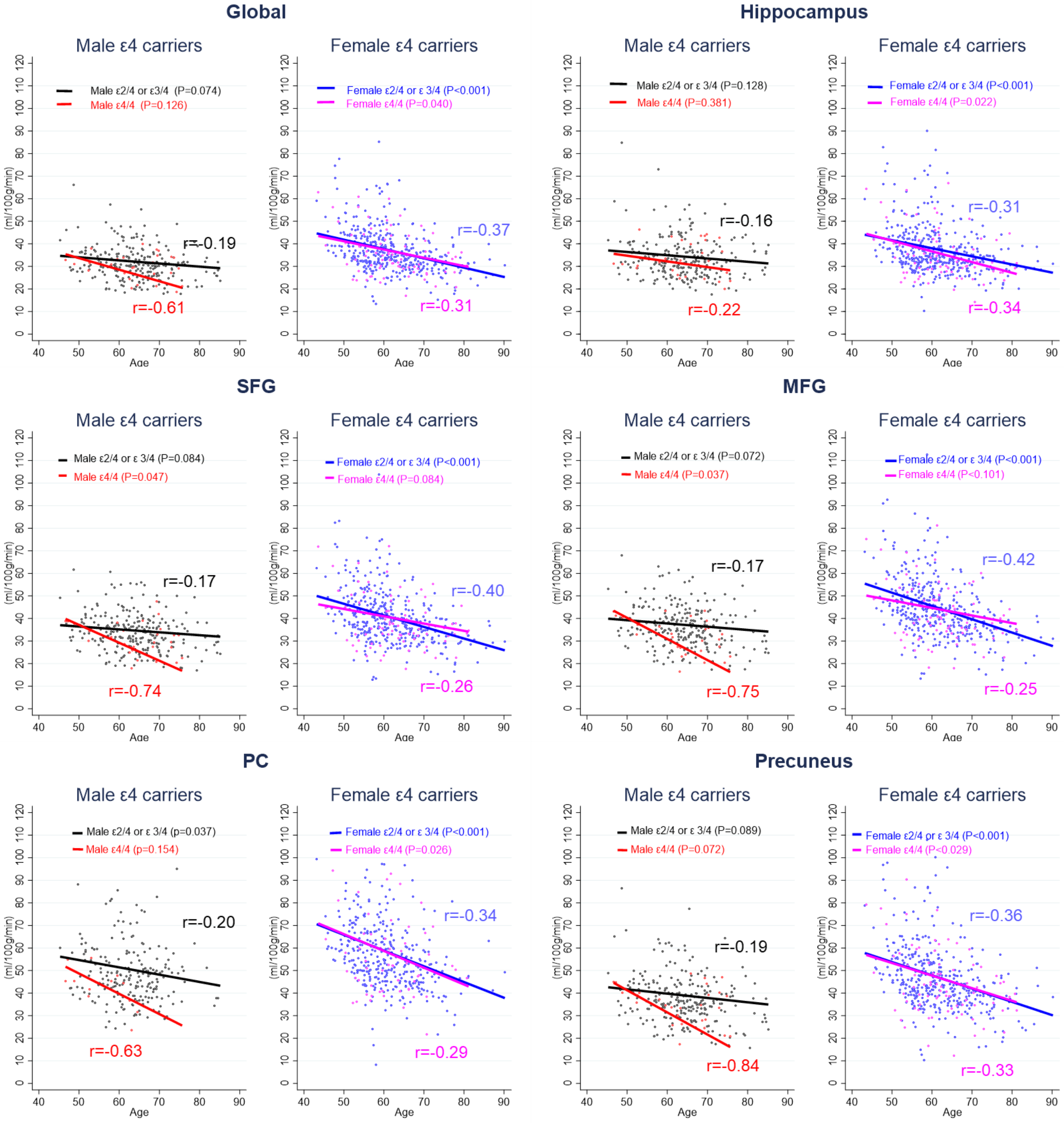

Supplementary Figure 1. Cerebral perfusion trajectories among *APOE* ε4 carriers by sex and *APOE* isoform

SFG=Superior frontal gyrus, MFG=middle frontal gyrus, PC=Posterior cingulate

Notes. There were 132 male ε2/4 or ε3/4 carriers, 225 female ε2/4 or ε3/4 carriers, 22 male ε4/4 carriers, and 46 female ε4/4 carriers.

Supplementary Table 1. Association between age-related cerebral perfusion change and time-varying cardiometabolic measurements

	**Gray matter**[a]	**Hippocampus**[a]	**Superior frontal gyrus**[a]	**Middle frontal gyrus**[a]	**Posterior cingulate**[a]	**Precuneus**[a]
Systolic blood pressure (n=881)	-0.08 (-0.10, -0.05)**	-0.08 (-0.12, -0.05)**	-0.08 (-0.11, -0.04)**	-0.09 (-0.13, -0.05)**	-0.13 (-0.19, -0.08)**	-0.09 (-0.13, -0.04)**
Body mass index (n=881)	-0.34 (-0.43, -0.25)**	-0.34 (-0.44, -2.06)**	-3.09 (-3.62, -2.56)**	-0.44 (-0.55, -0.32)**	-0.58 (-0.76, -0.41)**	-0.47 (-0.61, -0.34)**
Blood glucose (n=822)	-0.07 (-0.10, -0.04)**	-0.06 (-0.10, -0.03)**	-0.09 (-0.12, -0.05)**	-0.10 (-0.13, -0.06)**	-0.12 (-0.18, -0.06)**	-0.11 (-0.15, -0.07)**
Total cholesterol (n=883)	-1.07 (-1.57, -2.02)**	-0.96 (-1.54, -0.38)**	-1.18 (-1.76, -0.59)**	0.00 (0.00, 0.01)	0.01 (0.00, 0.02)	0.01 (0.00, 0.01)

[a] β-coefficients and 95% confidence intervals in models were adjusted for birth cohort, sex, APOE ε4 status, education, parental history of dementia, smoking status, intracranial volume, post-labeling delay, and head coil.

†0.05<*P*<0.1; *0.01<*P*<0.05; ***P*<0.01.

Supplementary Table 2. Association of cerebral perfusion with age, sex, and *APOE* ε4 status among participants with a 2025 ms post-labeling delay cerebral perfusion (n=876)

	Total Gray matter[a]	Hippocampus[a]	Superior frontal gyrus[a]	Middle frontal gyrus[a]	Posterior cingulate[a]	Precuneus[a]
Linear model, 5-yrs						
Age, 5-year	-1.11** (-1.47, -0.74)	-0.98** (-1.35, -0.61)	-1.32** (-1.74, -0.91)	-1.54** (-2.01, -1.07)	-2.46** (-3.26, -1.67)	-2.14** (-2.76, -1.62)
Categorical age						
Age groups						
40-49	Ref.	Ref.	Ref.	Ref.	Ref.	Ref.
50-59	-2.84** (-4.66, -1.02)	-3.00** (-5.06, -0.95)	-2.80** (-4.78, -0.82)	-3.01** (-5.15, -0.87)	-5.76** (-9.16, -2.36)	-4.95** (-7.63, -2.26)
60-69	-3.82** (-5.86, -1.78)	-3.89** (-6.14, -1.63)	-4.26** (-6.50, -2.01)	-4.59** (-7.05, -2.13)	-7.99** (-12.00, -3.99)	-7.40** (-10.57, -4.23)
≥70	-5.69** (-8.09, -3.28)	-5.61** (-8.20, -3.02)	-6.28** (-8.99, -3.58)	-6.96** (-9.96, -3.96)	-11.68** (-16.66, -6.69)	-10.46** (-14.43, -6.50)
Annual change rate of cerebral perfusion (Slope)						
Male *APOE non*-ε4	-1.44** (-2.07, -0.80)	-1.35** (-2.02, -0.67)	-1.60** (-2.30, -0.89)	-1.92** (-2.71, -1.13)	-2.83** (-4.19, -1.46)	-2.33** (-3.39, -1.26)
Male *APOE* ε4	-0.56 (-1.38, 0.27)	-0.35 (-1.23, 0.53)	-0.77† (-1.68, 0.14)	-0.85† (-1.87, 0.17)	-1.47 (-3.14, 0.20)	-0.96 (-2.26, 0.34)
Female *APOE non*-ε4	-1.16** (-1.66, -0.67)	-1.01** (-1.52, -0.49)	-1.33** (-1.88, -0.77)	-1.55** (-2.17, -0.93)	-2.41** (-3.47, -1.35)	-2.30** (-3.12, -1.47)
Female *APOE* ε4	-1.97** (-3.13, -0.81)	-1.91** (-3.15, -0.67)	-2.27** (-3.55, -1.00)	-2.79** (-4.21, -1.36)	-4.37** (-6.77, -1.98)	-4.00** (-5.86, -2.13)

[a]The β-coefficients and 95% confidence intervals in the models were adjusted for birth cohort, sex, *APOE* ε4 status, education, parental history of dementia, smoking status, intracranial volume, and head coil. †0.05<*P*<0.1; *0.01<*P*<0.05; ***P*<0.01.

Supplementary Table 3. Non-linear associations of cerebral perfusion with age, sex, and *APOE* ε4 (n=950)

	**Gray matter**[a]	**Hippocampus**[a]	**Superior frontal gyrus**[a]	**Middle frontal gyrus**[a]	**Posterior cingulate**[a]	**Precuneus**[a]
Quadratic model						
Age, year	-1.47**	-1.61**	-1.56**	-1.61**	-2.75**	-2.20**
	(-2.29, -0.66)	(-2.54, -0.67)	(-2.51, -0.60)	(-2.63, -0.59)	(-4.32, -1.18)	(-3.39, -1.00)
Age square	0.01*	0.01*	0.01†	0.01†	0.01*	0.01†
	(0.00, 0.01)	(0.00, 0.02)	(-0.00, 0.01)	(0.00, 0.01)	(0.00, 0.03)	(0.00, 0.02)
**Interaction, Sex**						
Quadratic model						
Age×Sex	-1.02	-0.48	-1.69†	-1.54	-1.88	-1.73
	(-2.65, 0.60)	(-2.33, 1.36)	(-3.59, 0.22)	(-3.58, 0.49)	(-5.01, 1.25)	(-4.11, 0.65)
Age square ×Sex	0.01	0.00	0.01	0.01	0.01	0.01
	(-0.01, 0.01)	(-0.01, 0.02)	(-0.00, 0.03)	(-0.01, 0.03)	(-0.01, 0.04)	(-0.01, 0.03)
**Interaction, *APOE* ε4**						
Quadratic model						
Age×ε4	-1.35†	-1.43	-1.37	-1.33	-2.95†	-1.81
	(-2.90, 0.20)	(-3.20, 0.34)	(-3.19, 0.45)	(-3.27, 0.62)	(-5.94, 0.04)	(-4.09, 0.46)
Age square ×ε4	0.01†	0.01	0.01	0.01	0.02*	0.02
	(-0.00, 0.02)	(-0.00, 0.03)	(-0.00, 0.03)	(0.00, 0.03)	(0.00, 0.05)	(0.00, 0.03)

[a] β-coefficients and 95% confidence intervals in models were adjusted for birth cohort, sex, APOE ε4 status, education, parental history of dementia, smoking status, intracranial volume, post-labeling delay, and head coil.

†0.05<*P*<0.1; *0.01<*P*<0.05; ***P*<0.01.

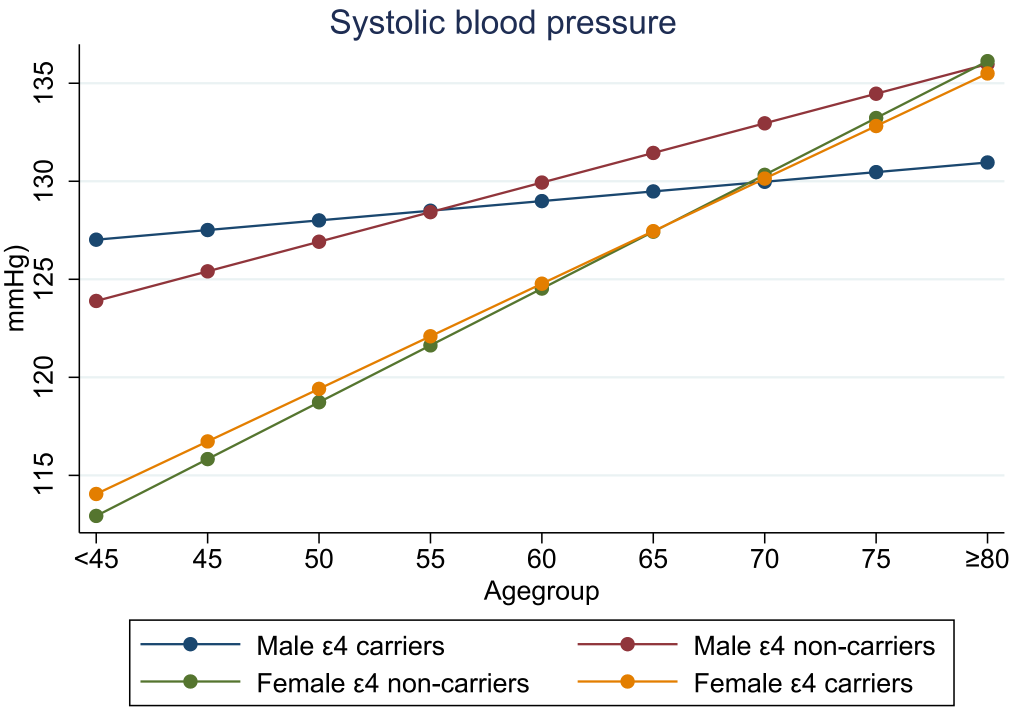

Supplementary Figure 2. The association between systolic blood pressure and age by *APOE* ε4 status and sex (n=876)
